# Denosumab compared to bisphosphonates to treat postmenopausal osteoporosis: a meta-analysis

**DOI:** 10.1186/s13018-018-0865-3

**Published:** 2018-08-02

**Authors:** Jiaqi Wu, Qingsheng Zhang, Guanghui Yan, Xianhui Jin

**Affiliations:** The Second Department of Orthepaedics, Harrison International Peace Hospital, No. 180 Renmin East Road, Hengshui, 053000 Hebei China

**Keywords:** Denosumab, Bisphosphonates, Fracture, Meta-analysis

## Abstract

**Background:**

The standard treatment for osteoporosis was controversial. Denosumab and bisphosphonates were two most common drugs. The purpose of this study was to compare the efficacy and safety of denosumab with bisphosphonates to treat osteoporosis.

**Methods:**

Published literatures, only including randomized controlled trials (RCTs), were searched in the following electronic databases: PubMed, Embase, Web of Science, Cochrane Library, and Google database from inception to April 20 2018. Studies that compared denosumab with bisphosphonates to treat osteoporosis were included. Random-effect model was used for meta-analysis due to the unavoidable clinical heterogeneity. We used the risk of fracture as the primary outcome. Stata 12.0 was used for meta-analysis.

**Results:**

Eleven studies involving 5446 patients (denosumab = 2873, bisphosphonates = 2573) were included in the present meta-analysis. There was no significant difference between the risk of fracture (risk ratio (RR), 1.13; 95% confidence interval (CI), 0.82–1.55; *P* = 0.466), adverse events (AEs) (RR 1.00; 95% CI 0.96–1.04; *P* = 0.957) and withdrawn due to AEs (RR 0.68; 95% CI 0.34–137; *P* = 0.280). Denosumab compared with bisphosphonates significantly increased change in total hip, femoral neck, lumbar spine, and one-third radius bone mineral density (BMD) for postmenopausal osteoporosis patients (*P* < 0.05).

**Conclusions:**

Our meta-analysis suggested that denosumab but not bisphosphonates significantly increased change in total hip, femoral neck, lumbar spine, and one-third radius BMD for postmenopausal osteoporosis patients. Current evidence suggested no benefit of denosumab for reducing risk of fracture than bisphosphonates. More long-term follow-up RCTs are needed to identify the potential complications of denosumab.

**Electronic supplementary material:**

The online version of this article (10.1186/s13018-018-0865-3) contains supplementary material, which is available to authorized users.

## Background

Osteoporosis (OP) is a common contributor to hip and spine fractures in worldwide patients [1, 2]. OP is a global public health problem and affects appropriately 75 million people in the USA [3]. The ideal therapeutic goal is to increase the bone mass, and subsequently decrease the risk of fracture [4]. Bisphosphonates, a classic antiresorptive agent, is currently the most common therapy for osteoporosis [5]. However, compliance was the major concern of bisphosphonates [6]. Prolonged medication and possible complications limited the effects of bisphosphonates for OP patients.

Receptor activator of nuclear factor-kB ligand (RANKL) is a cytokine that is essential for osteoclast survival and differentiation. Thus, through blocking the RANKL could inhibit the differentiation of osteoclast and increase the bone mass. Denosumab, a fully human monoclonal antibody against the RANKL, could potently reduce bone resorption with accompanying increases in bone mineral density (BMD). Denosumab 60 mg was always subcutaneously given per 6 months and thus the compliances may be well. Previously, a meta-analysis that compared denosumab with bisphosphonates for OP patients has been published [7]. However, several disadvantages existed in the meta-analysis. (1) Only four randomized controlled trials (RCTs) were included and the sample was relatively small. (2) They only compared with alendronate, but neglect other bisphosphonates. With new evidence emerging, we performed a meta-analysis that compares denosumab with bisphosphonates for bone loss in postmenopausal osteoporosis patients. We hypothesized that denosumab was superior than bisphosphonates in reducing bone loss in postmenopausal osteoporosis patients and thus reduce the risk of fracture.

## Methods

The present meta-analysis was performed according to the Preferred Reporting Items for Systematic Reviews and Meta-Analyses statement (PRISRMA) [8].

### Search strategy

Published literatures, only including RCTs, were searched in the following electronic databases: PubMed, Embase, Web of Science, Cochrane Library, and Google database from inception to April 20 2018. The keywords and corresponding Mesh terms of osteoporosis and denosumab were referred to published meta-analysis [9]. The keywords and corresponding Mesh term of bisphosphonates was referred to Lou’s protocol [10]. Detailed search keywords and Mesh terms can be seen in Additional file 1. Besides, references of all included articles were also reviewed. There was no language limited and no publication restriction.

### Inclusion criteria and exclusion criteria

#### Inclusion criteria

Candidate articles would be included if they met the following criteria: (i) population: patients were diagnosed with osteoporosis and did not take other oral anti-osteoporosis drugs; (ii) intervention: subcutaneously administered denosumab 60 mg per 6 months; (iii) comparison: administration with bisphosphonates, regardless of the dose and intervals; (iv) with one or more of the outcomes described below: risk of fracture, total adverse events (AEs), withdrawn due to AEs, change in one-third radius BMD, change in total hip BMD, change in lumbar spine BMD, and change in femoral neck BMD; and (v) study design: RCTs.

#### Exclusion criteria

Candidate articles would be excluded if they met the following criteria: (i) patients were diagnosed with other type of osteoporosis (androgen-deprivation therapy or breast cancer); (ii) intervention: combined denosumab with other drugs; and (iii) non-RCTs.

### Assessment of study quality

Two reviewers (Jiaqi Wu and Qingsheng Zhang) independently assessed the quality of RCTs in accordance with Cochrane Collaboration’s tool for assessing the risk of bias. If there was conflict between the two reviewers, a third reviewer is consulted and they discussed to solve the controversy. The tool included the following items: random sequence generation, allocation concealment, blinding, incomplete outcome data, and selective outcome reporting. We further performed Kappa test to increase the stability of our meta-analysis.

### Data extraction

Two reviewers independently extracted the following data and written in a pre-generated Excel file: first author’s name, publication year, mean age, intervention, comparison, outcomes, and duration. First outcome was the risk of fracture, since fracture has a heavy economic burden to the society and patients. Second outcomes were change in one-third radius BMD, change in total hip BMD, change in lumbar spine BMD, and change in femoral neck BMD. Safety outcomes included total AEs, withdrawn due to AEs.

### Statistical analysis

In consideration of the clinical heterogeneity, we used random-effect model for all of the outcomes. Outcomes were divided into two categories (dichotomous data and continuous data). Dichotomous data were expressed as proportions, such as risk of fracture, AEs, and withdrawn due to AEs; the intervention effect was expressed as a risk ratio (RR) and corresponding confidence intervals (CI). Continuous data were meta-analyzed in terms of the weighted mean difference (WMD) and associated 95% CI. Publication bias was assessed by funnel plot, Begg’s test, and Egger’s test. Subgroup analysis was performed according to the comparator treatment, population who had been prescribed a treatment for osteoporosis and high or unclear risk of bias.

Pooled data were assessed for heterogeneity using the *I*^2^ tests. Heterogeneity was defined as absent when *I*^2^ was between 0 and 25%; low, between 25.1 and 50%; moderate, between 50.1 and 75%; or high, between 75.1 and 100%.

## Result

### Search results

**Fig. 1:**
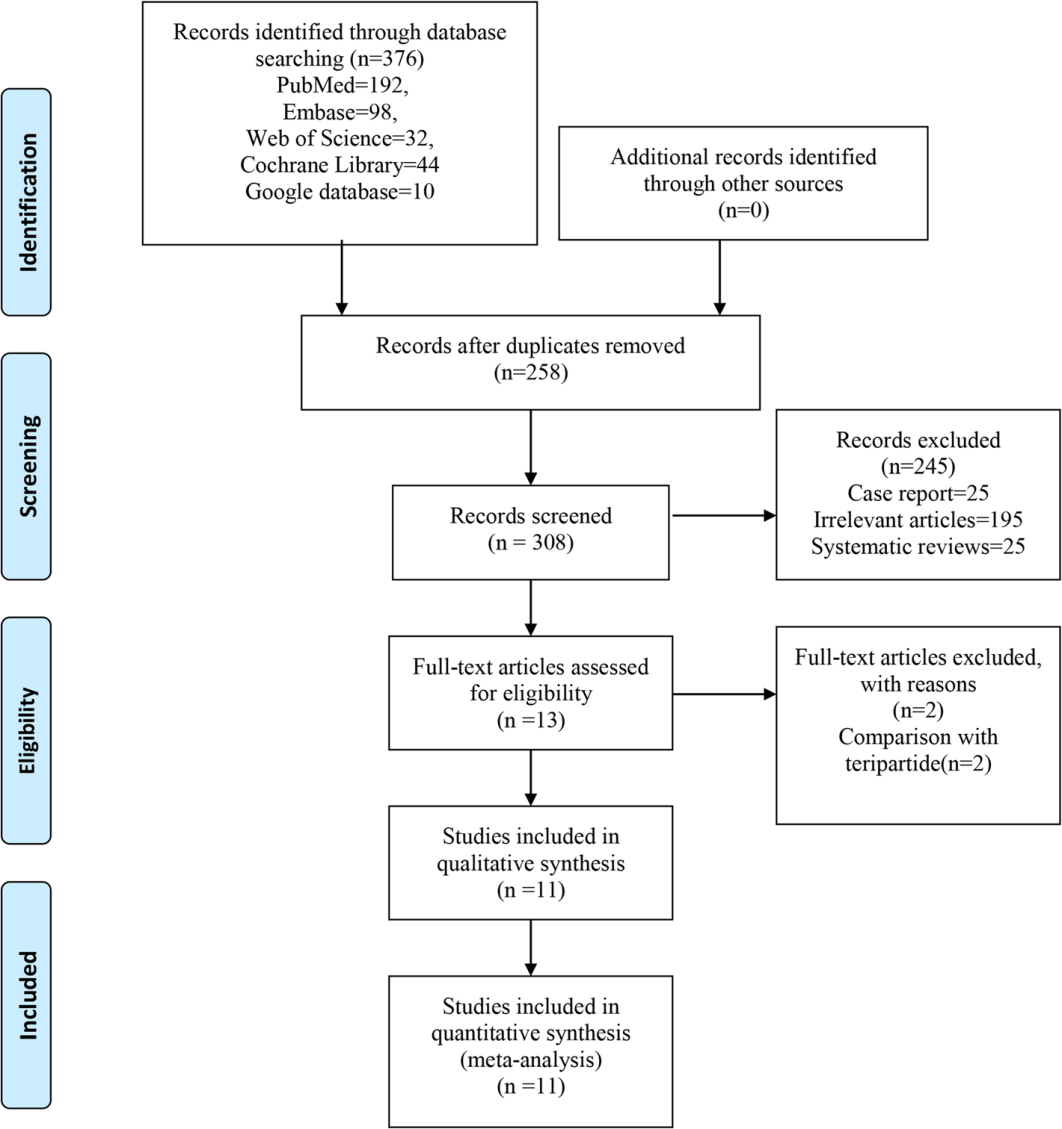
Flow of trials through the meta-analysis

Systematic search of PubMed, Embase, Web of Science, Cochrane Library, and Google database turned up 376 potentially eligible studies, and no additional records were found during manual searches of reference lists. After removing 118 duplicate studies using Endnote X7, another 245 studies were excluded based on their titles and abstracts. The remaining 13 studies were read in full, and 2 were excluded because they failed to satisfy the selection criteria. In the end, 11 RCTs involving 5446 patients (denosumab = 2873, bisphosphonates = 2573) [11–21] were included in the systematic review and meta-analysis. Details of study identification, screening, and selection are given in Fig. 1.

### Characteristics of included studies

The publication years were raged from the year of 2006 to 2016. Altogether involving 5449 cases of osteoporosis. Six studies were performed in the USA, 1 in Spain, 1 in UK, 2 in Canada, and 1 France and 1 Australia. The dose of denosumab was administration with subcutaneous injection 60 mg every 6 months. We included four types of bisphosphonates (alendronate, ibandronate, risedronate, and zoledronic acid). The duration of follow-up was ranged from 12 to 24 months. Detailed information of the characteristic can be obtained in Table 1.

**Table 1:** The general characteristic of the included RCTs

Author	Sample (*n*)			Mean age (year)		Intervention	Comparison	Duration (month)	Outcomes	Study
	Country	Intervention	Comparison	Intervention	Comparison					
Beck 2008 [11]	USA	39	38	63	63	Sc denosumab injections (60 mg Q6M)	Oral alendronate 70 mg once weekly	24	56	RCTs
Lewiecki 2007 [12]	USA	319	47	62.3	62.8	Sc denosumab injections (60 mg Q6M)	Oral alendronate 70 mg once weekly	24	1	RCTs
McClung 2006 [13]	USA	47	47	63.1	62.8	Sc denosumab injections (60 mg Q6M)	Oral alendronate 70 mg once weekly	12	123,467	RCTs
Brown 2009 [14]	Spain	594	595	64.1	64.6	Sc denosumab injections (60 mg Q6M)	Oral alendronate 70 mg once weekly	12	1,234,567	RCTs
Kendler 2010 [15]	USA	253	251	66.9	68.2	Sc denosumab injections (60 mg Q6M)	Oral alendronate 70 mg once weekly	12	234,567	RCTs
Freemantle 2012 [16]	UK	126	124	65.1	65.3	Sc denosumab injections (60 mg Q6M)	Oral alendronate 70 mg once weekly	24	2345	RCTs
Kendler 2011 [17]	Canada	253	251	66.9	68.2	Sc denosumab injections (60 mg Q6M)	Oral alendronate 70 mg once weekly	12	56	RCTs
Recknor 2013 [18]	USA	417	416	67.2	66.2	Sc denosumab injections (60 mg Q6M)	Oral ibandronate 150 mg once month	12	12,346	RCTs
Roux 2014 [19]	France	422	402	67.8	67.7	Sc denosumab injections (60 mg Q6M)	Oral risedronate 150 mg once month	12	123,456	RCTs
Miller 2016 [20]	USA	320	320	65.9	66.1	Sc denosumab injections (60 mg Q6M)	intravenous zoledronic acid 5 mg once year	12	123,457	RCTs
Seeman 2010 [21]	Australia	83	82	60.3	60.7	Sc denosumab injections (60 mg Q6M)	oral alendronate 70 mg once weekly	12	23	RCTs

1, risk of fracture; 2, AEs, 3 withdrawn due to AEs; 4, change in total hip BMD; 5, change in femoral neck BMD; 6, change in lumbar spine BMD; 7, change in one-third radius BMD

### Quality of the included RCTs

**Fig. 2:**
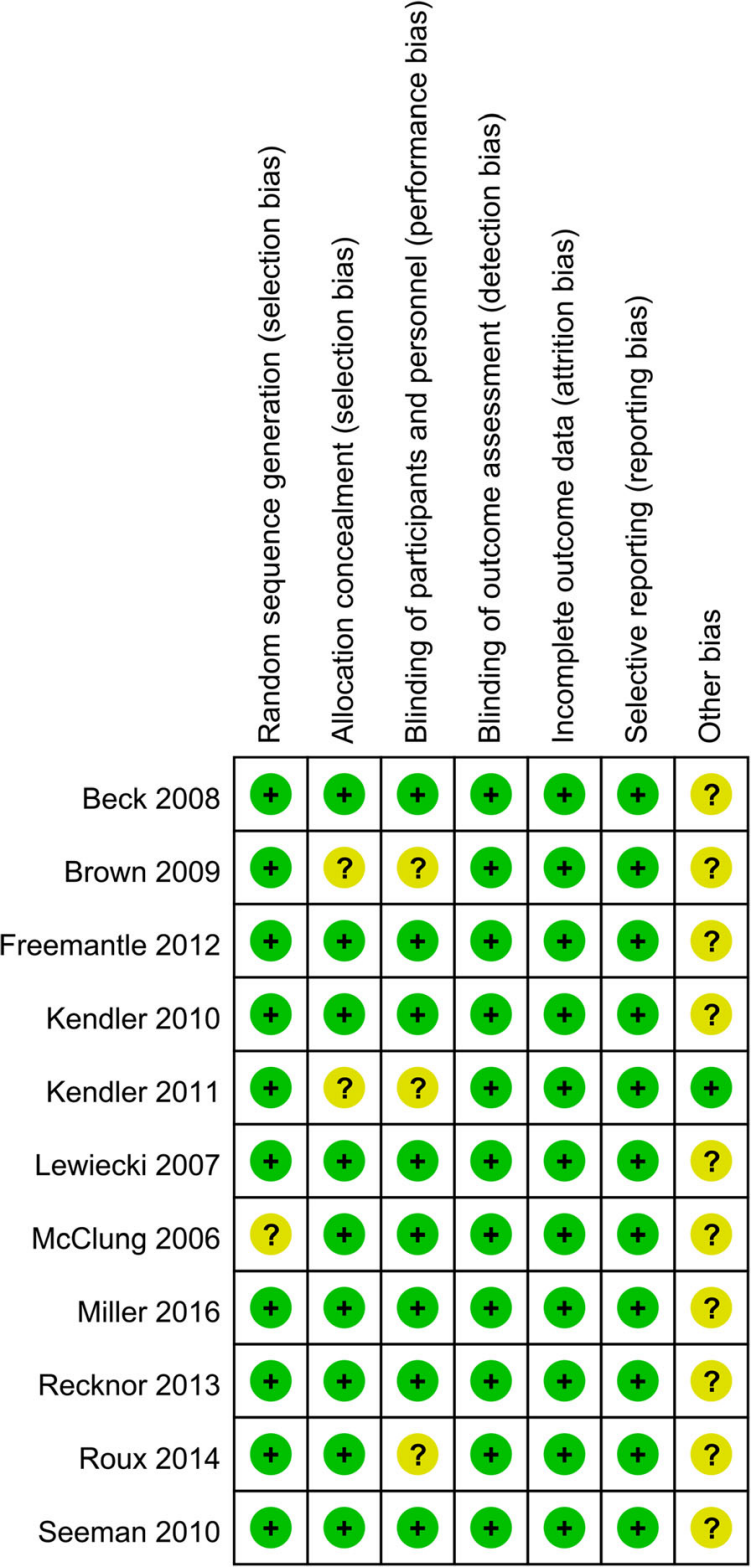
The risk of bias summary, +, no bias; −, bias;?, bias unknown

**Fig. 3:**
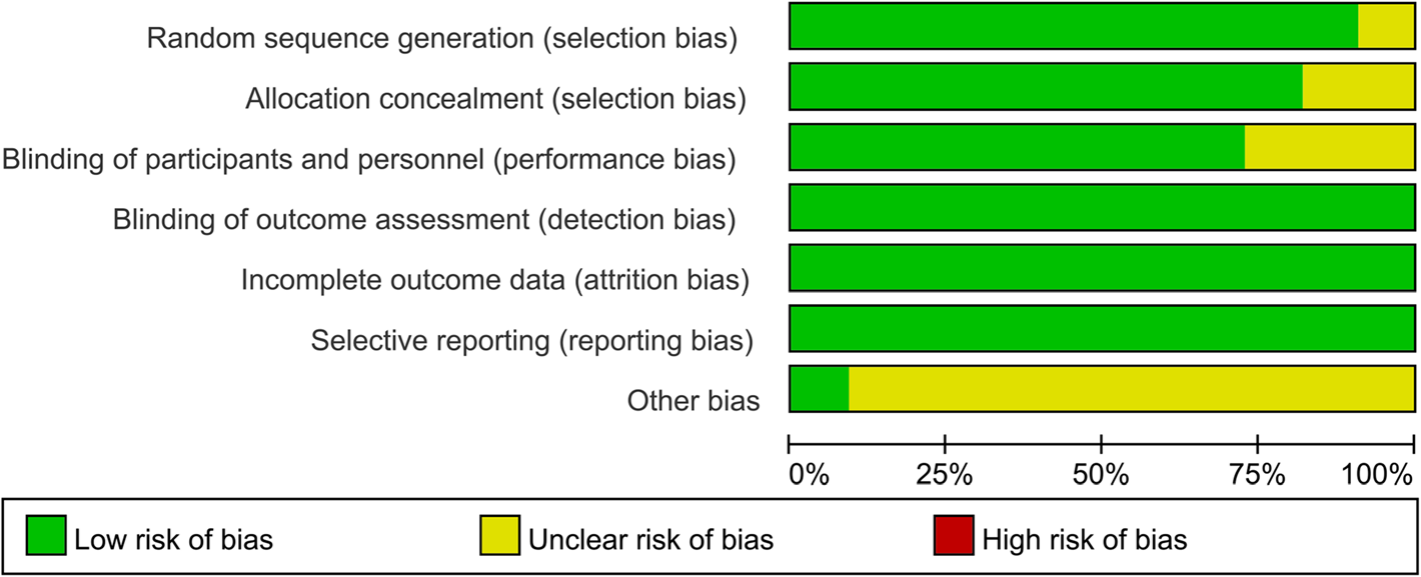
Risk of bias of graph of the included randomized controlled trials

Details of the risk of bias are summarized in Figs. 2 and 3. Overall, ten trials were categorized as at low risk of bias, one as being unclear, and none being at high risk of bias. Only one study did not introduce the random sequence generation and thus categorized as unclear risk of bias. Since we did not know whether the funding institution participated into the experimentation and thus all of the other bias was categorized as unclear risk of bias.

### Results of meta-analysis

#### Risk of fracture

**Fig. 4:**
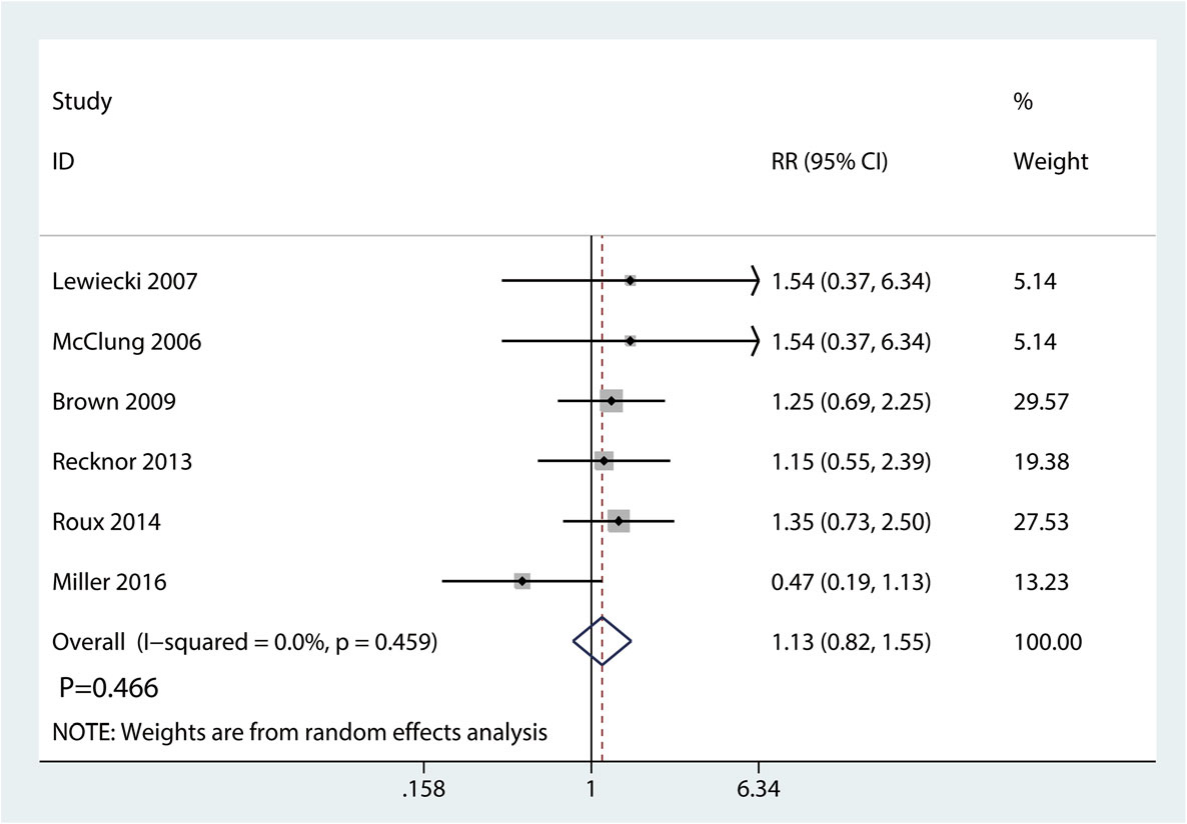
Forest plots of the included studies comparing the risk of fracture

**Fig. 5:**
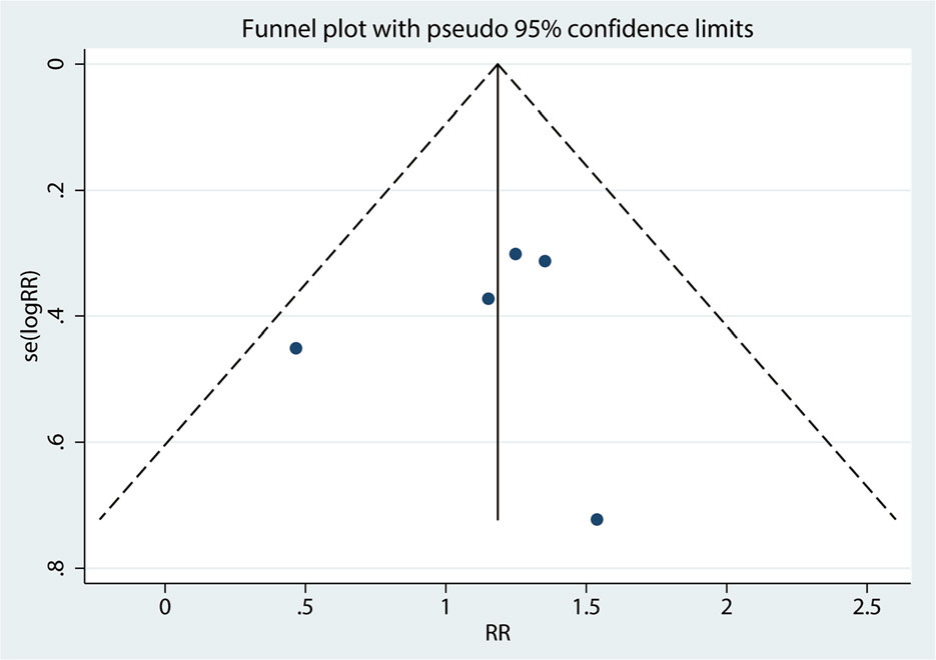
Funnel plot of the risk of fracture

**Fig. 6:**
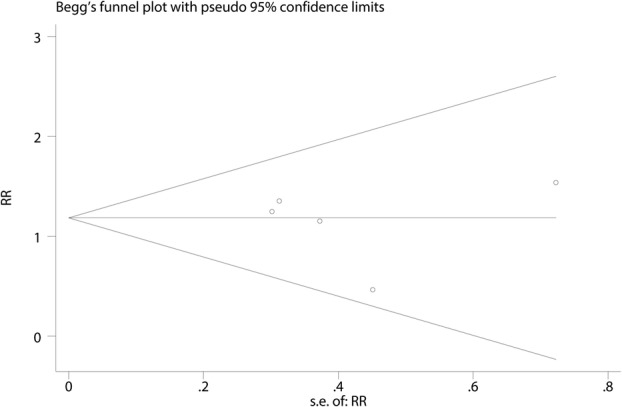
Egger test for risk of fracture

**Fig. 7:**
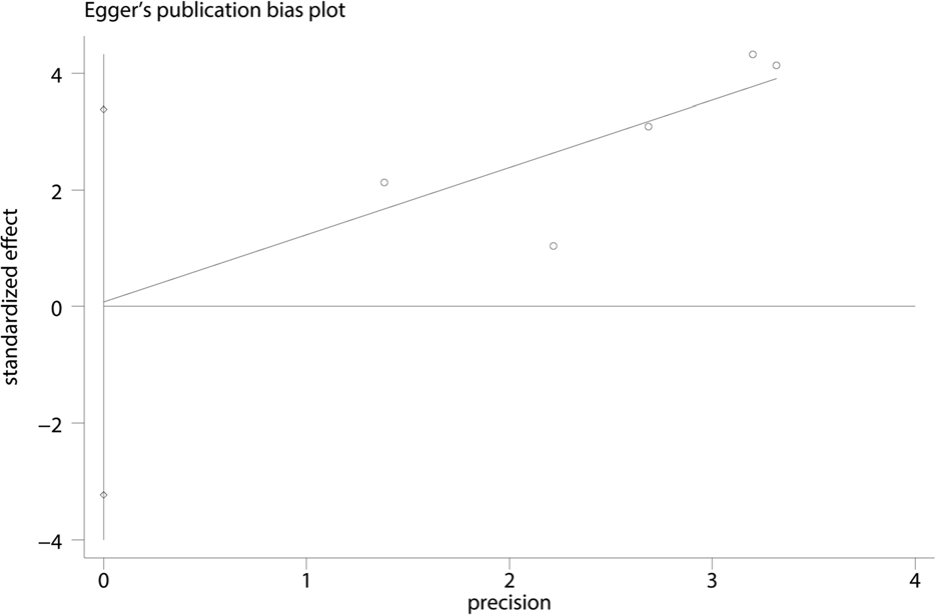
Begg’s test for risk of fracture

**Fig. 8:**
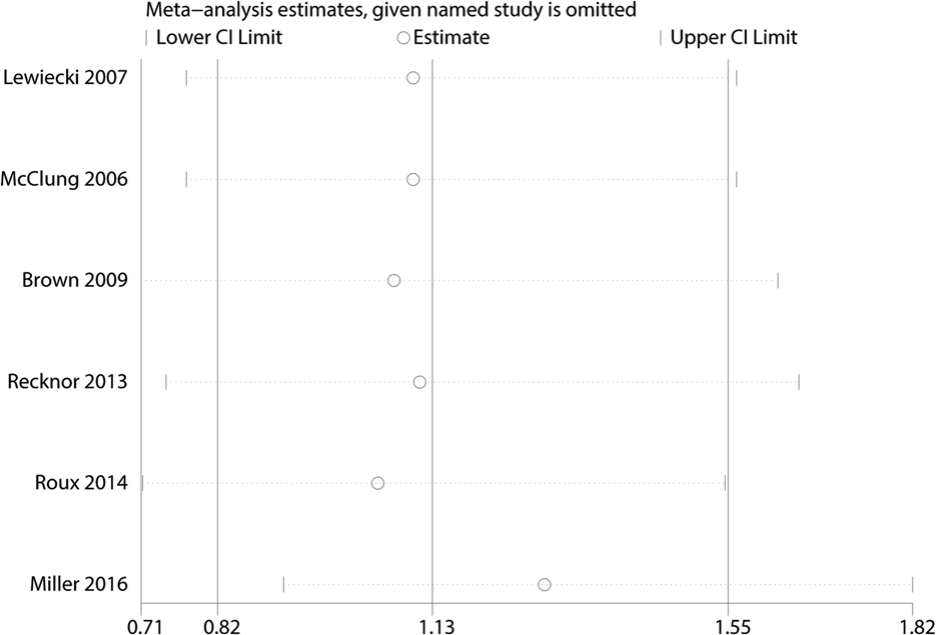
Sensitivity analysis for risk of fracture

Six trials totaling 4218 patients provided data on risk of fracture. Compared with bisphosphonates treatment, administration with denosumab has no benefit for reducing the risk of fracture (RR 1.13; 95% CI 0.82–1.55; *P* = 0.466) (Fig. 4), with no heterogeneity (*I*^2^ = 0.0%). For the meta-analysis of denosumab versus bisphosphonates on risk of fracture, there was no evidence of publication bias by inspection of the funnel plot (Fig. 5) and formal statistical tests (Egger test, *P* = 0.85; Begg test, *P* = 0.69) **(**Figs. 6 and 7). To further increase the persuasion of this outcome, we performed a sensitivity analysis using leave out one study in turn and results shown that after leaving out one study in turn, the overall effect size was not changeable (Fig. 8).

Table 2 presents the results of subgroup analyses. The findings of decreased risk of fracture were consistent in all subgroup analyses.

**Table 2:** Subgroup analysis of the risk of fracture

Subgroup	No. of included studies	RR (95% CI)	*I*^2^ (%)
Comparator treatment			
Alendronate	3	1.10 (0.95, 1.23)	12.3
Ibandronate	1	1.08 (0.89, 1.16)	–
Risedronate	1	1.23 (0.75, 1.08)	–
Zoledronic acid	1	0.86 (0.74, 0.99)	–
Population who had been prescribed a treatment for osteoporosis			
No (< 100% of participants)	4	1.15 (0.67, 1.22)	0.0
No (> 100% of participants)	3	1.12 (0.85, 1.08)	0.0
High or unclear risk of bias			
No	2	1.09 (0.74, 1.26)	0.0
Yes	5	1.31 (0.89, 1.14)	0.0

### AEs

**Fig. 9:**
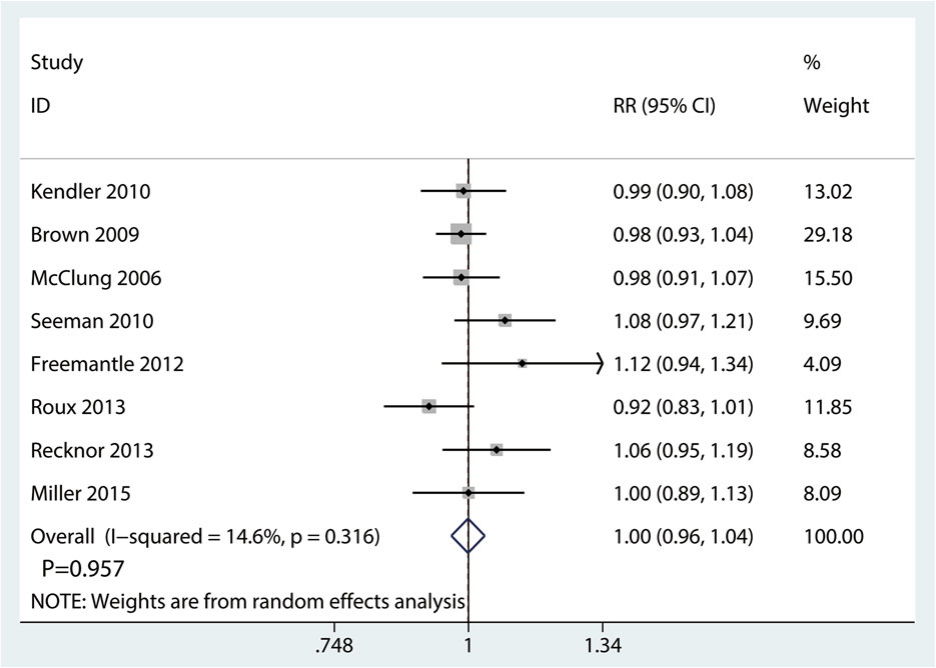
Forest plots of the included studies comparing the AEs

Seven trials totaling 4776 patients provided data on AEs. There was no significant difference between denosumab and bisphosphonates in terms of the AEs (RR 1.00; 95% CI 0.96–1.04; *P* = 0.957) (Fig. 9), with little heterogeneity (*I*^2^ = 14.6%).

### Withdrawn due to AEs

**Fig. 10:**
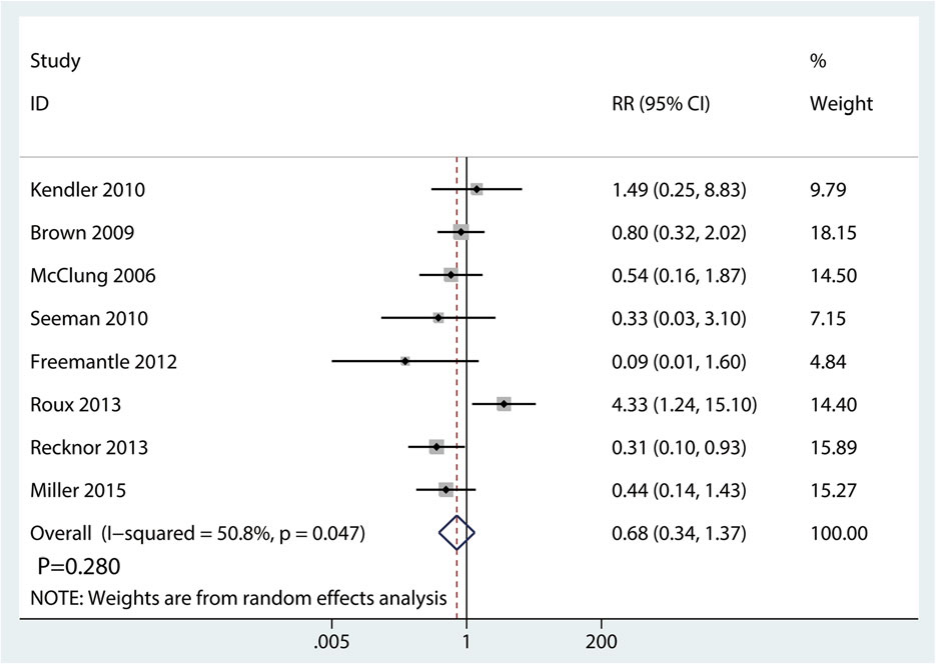
Forest plots of the included studies comparing the withdrawn due to AEs

Eight trials totaling 4816 patients provided data on withdrawn due to AEs. There was no significant difference between denosumab and bisphosphonates in terms of the withdrawn due to AEs (RR 0.68; 95% CI 0.34–137; *P* = 0.280) (Fig. 10), with middle heterogeneity (*I*^2^ = 50.8%).

### Change in total hip BMD

**Fig. 11:**
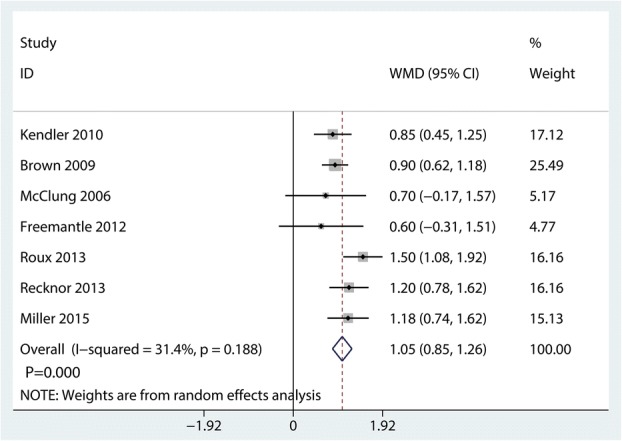
Forest plots of the included studies comparing the change in total hip BMD

Compared with bisphosphonates, denosumab for osteoporosis patients further increased change in total hip BMD (MD 1.05%; 95% CI 0.85 to 1.26; *P* = 0.000, Fig. 11) with middle heterogeneity (*I*^2^ = 31.4%).

### Change in femoral neck BMD

**Fig. 12:**
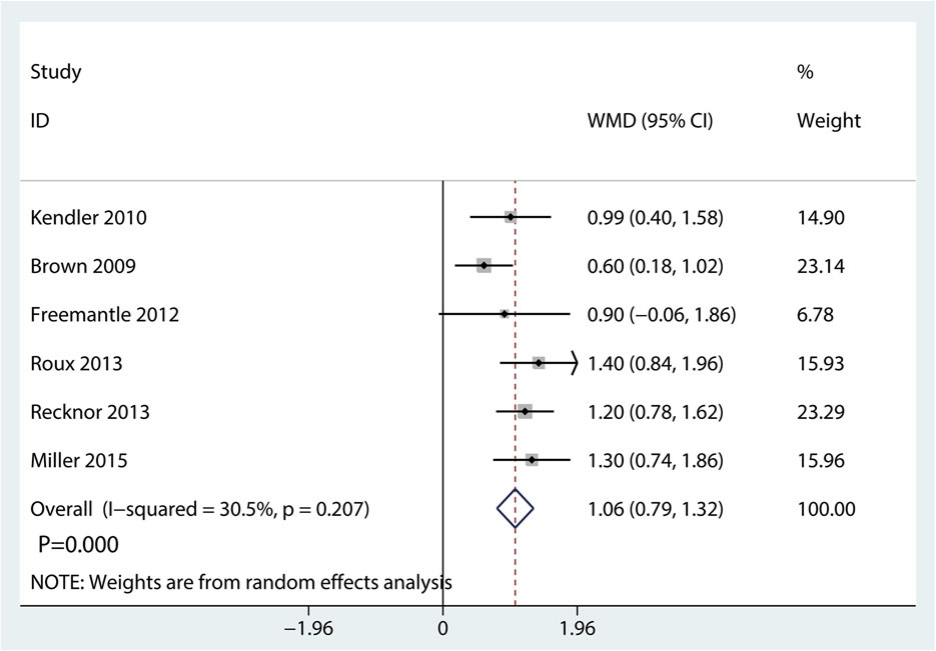
Forest plots of the included studies comparing the change in femoral neck BMD

Compared with bisphosphonates, denosumab for osteoporosis patients further increased change in femoral neck BMD, (WMD = 1.06%; 95% CI 0.79 to 1.32; *P* = 0.000, Fig. 12) with middle heterogeneity (*I*^2^ = 30.5%).

### Change in lumbar spine BMD

**Fig. 13:**
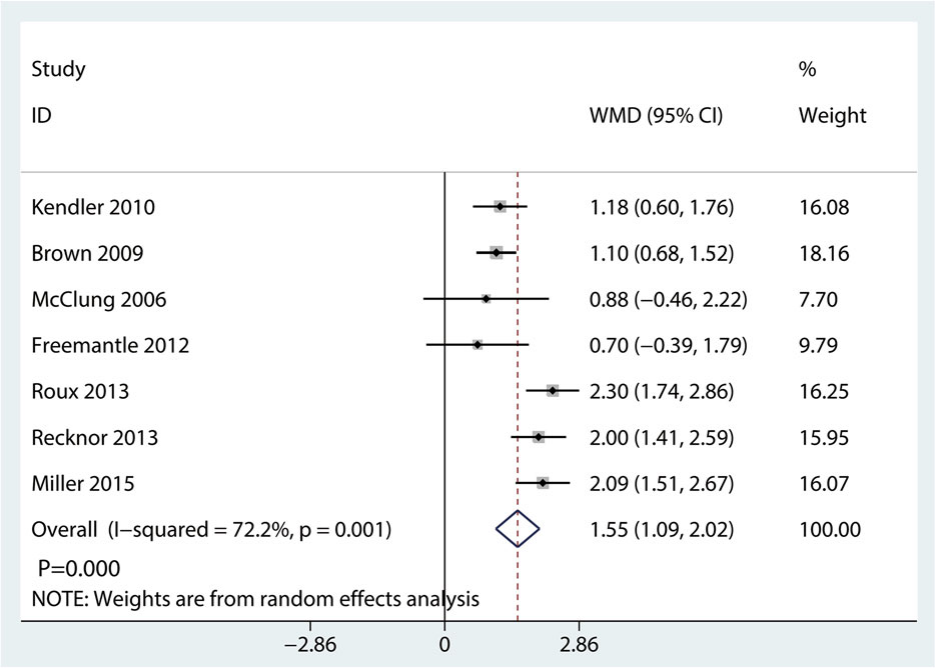
Forest plots of the included studies comparing the change in lumbar spine BMD

Compared with bisphosphonates, denosumab for osteoporosis patients further increased change in lumbar spine BMD (WMD = 1.55%; 95% CI 1.09 to 2.02; *P* = 0.000, Fig. 13) with high heterogeneity (*I*^2^ = 72.2%).

### Change in one-third radius BMD

**Fig. 14:**
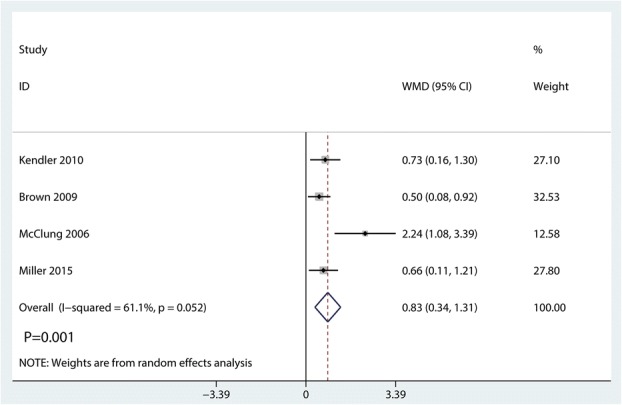
Forest plots of the included studies comparing the change in one-third radius BMD

Compared with bisphosphonates, denosumab for osteoporosis patients further increased change in one-third radius BMD (WMD = 0.83%; 95% CI 0.34 to 1.31; *P* = 0.000, Fig. 14) with high heterogeneity (*I*^2^ = 61.1%).

## Discussion

Our meta-analysis comprehensively and systematically reviewed the current available literature and found that (1) denosumab compared with bisphosphonates significantly increased change in total hip, femoral neck, lumbar spine, and one-third radius BMD for postmenopausal osteoporosis patients. The evidence of benefit was consistent when we further performed a sensitivity analysis; (2) denosumab has no benefit for reducing the risk of fracture when compared with bisphosphonates; (3) and the occurrence of AEs and withdrawn due to AEs were similar in denosumab and bisphosphonates.

Several meta-analyses on the topic have been published [7, 9]. However, differences between current meta-analysis and the previous ones should be noted. First, these previous meta-analyses identified other anti-osteoporosis drugs (teriparatide) and placebo as comparison. Therefore, a large heterogeneity existed in previous meta-analyses. Second, several literatures were left out in previous meta-analyses and thus potential publication bias existed in their study. Beaudoin et al. [9] compared denosumab with other treatments to prevent or treat osteoporosis. We noted that they included patients that prescribed daily or weekly bisphosphonate therapy ≥ 1 month. Thus, we could not determine the real effects of denosumab for postmenopausal osteoporosis. Lin et al. [7] conducted a meta-analysis that compared denosumab and alendronate in postmenopausal women with osteoporosis. However, only four heterogeneous RCTs were included. Current meta-analysis added statistical power of at least 7 RCTs and appropriately 1900 cases. In summary, our current meta-analysis was the latest and the most comprehensive one.

The difference between denosumab and bisphosphonates for clinical outcomes were change in the skeletal BMD. Denosumab has a greater antiresorptive effect than bisphosphonates. The effects of bisphosphonates for preventing bone loss mainly need bisphosphonates binding to bone mineral. Denosumab mainly through direct combined with the RANKL and inhibit the survival and differentiation of osteoclast. In general, increasing of the BMD means a decrease in the occurrence of fracture. However, we did not observe any significant difference between the risk of fracture. The reason may be as follows: (1) gain in BMD over a relatively short time was not enough to reduce the risk of fracture, and (2) the occurrence of fracture was affected by many factors. Murad et al. [22] conducted a network meta-analysis and compared with different drugs for preventing fragility fractures. Results show that teriparatide, bisphosphonates, and denosumab are the most effective in reducing the risk of fragility fractures.

When a new drug was popularized and applied in clinical trials, AEs were the major concern. In current meta-analysis, we compared total AEs and withdrawn due to AEs. Results show that there was no significant difference between the total AEs and withdrawn due to AEs. Since immune cells also have the RANKL receptor and the major concern was the denosumab for immune function. Later, Stolina et al. [23] and Bekker et al. [24] suggest that denosumab has no effects on RANKL/RANK pathway in immune system.

Our meta-analysis also had limitations. (1) Included studies were sponsored by drug dealer and thus may resulted in performance bias. (2) We included osteoporosis patients that came from different countries and with different diagnostic criteria. Thus, the clinical heterogeneity was imminent. (3) Duration of follow-up was relatively short in the included studies and thus some severe complications were underestimated.

## Conclusion

Our meta-analysis suggested that denosumab but not bisphosphonates significantly increased change in total hip, femoral neck, lumbar spine, and one-third radius BMD for postmenopausal osteoporosis patients. Current evidence suggested no benefit of denosumab for reducing risk of fracture than bisphosphonates. More long-term follow-up RCTs are needed to identify the potential complications of denosumab.

## Additional file

Additional file 1: Detailed search keywords and Mesh terms in PubMed database. (DOCX 14 kb)

## Abbreviation

AEs: Adverse events
BMD: Bone mineral density
CI: Confidence intervals
OP: Osteoporosis
RANKL: Receptor activator of nuclear factor-kB ligand
RCTs: Randomized controlled trials
RR: Risk ratio
WMD: Weighted mean difference
