# Supplementary material for: Denosumab compared to bisphosphonates to treat postmenopausal osteoporosis: a meta-analysis
Source: J Orthop Surg Res. 2018 Aug 2;13:194. doi: 10.1186/s13018-018-0865-3 (PMC6090940; doi:10.1186/s13018-018-0865-3)
Supplement: Supplementary file 1 — Detailed search keywords and Mesh terms in PubMed database. (DOCX 14 kb) [file 13018_2018_865_MOESM1_ESM.docx]

Search key words and Mesh terms for denosumab:

Xgeva

AMG 162

Prolia

Denosumab

"Denosumab"[Mesh]

Search key words and Mesh terms for bisphosphonates:

Bisphosphonates

Alendronate

Clodronic Acid

Etidronic Acid

Risedronate Sodium

Technetium Tc 99m Medronate

"Diphosphonates"[Mesh]

Search key words and Mesh terms for osteoporosis:

Perimenopausal Bone Loss

Bone Loss, Postmenopausal

Bone Losses, Postmenopausal

Postmenopausal Bone Losses

Osteoporosis, Post-Menopausal

Osteoporoses, Post-Menopausal

Osteoporosis, Post Menopausal

Post-Menopausal Osteoporoses

Post-Menopausal Osteoporosis

Postmenopausal Osteoporosis

Osteoporoses, Postmenopausal

Postmenopausal Osteoporoses

Bone Loss, Perimenopausal

Bone Losses, Perimenopausal

Perimenopausal Bone Losses

Postmenopausal Bone Loss

"Osteoporosis, Postmenopausal"[Mesh]
